# Genomic variation in the American pika: signatures of geographic isolation and implications for conservation

**DOI:** 10.1186/s12862-020-01739-9

**Published:** 2021-01-21

**Authors:** Kelly B. Klingler, Joshua P. Jahner, Thomas L. Parchman, Chris Ray, Mary M. Peacock

**Affiliations:** 1 Department of Biology, University of Nevada, Reno, 89557 USA; 2 Program in Ecology, Evolution, and Conservation Biology, University of Nevada, Reno, NV 89557 USA; 3 Department of Ecology and Evolutionary Biology, University of Colorado, Boulder, CO 80309-0334 USA

**Keywords:** Alpine, Climate, Conservation, Genetic diversity, Genotyping-by-sequencing, Great basin, Metapopulation, *Ochotona princeps*, Rocky Mountains, Sierra Nevada

## Abstract

**Background:**

Distributional responses by alpine taxa to repeated, glacial-interglacial cycles throughout the last two million years have significantly influenced the spatial genetic structure of populations. These effects have been exacerbated for the American pika (*Ochotona princeps*), a small alpine lagomorph constrained by thermal sensitivity and a limited dispersal capacity. As a species of conservation concern, long-term lack of gene flow has important consequences for landscape genetic structure and levels of diversity within populations. Here, we use reduced representation sequencing (ddRADseq) to provide a genome-wide perspective on patterns of genetic variation across pika populations representing distinct subspecies. To investigate how landscape and environmental features shape genetic variation, we collected genetic samples from distinct geographic regions as well as across finer spatial scales in two geographically proximate mountain ranges of eastern Nevada.

**Results:**

Our genome-wide analyses corroborate range-wide, mitochondrial subspecific designations and reveal pronounced fine-scale population structure between the Ruby Mountains and East Humboldt Range of eastern Nevada. Populations in Nevada were characterized by low genetic diversity (π = 0.0006–0.0009; θ_W_ = 0.0005–0.0007) relative to populations in California (π = 0.0014–0.0019; θ_W_ = 0.0011–0.0017) and the Rocky Mountains (π = 0.0025–0.0027; θ_W_ = 0.0021–0.0024), indicating substantial genetic drift in these isolated populations. Tajima’s *D* was positive for all sites (*D* = 0.240–0.811), consistent with recent contraction in population sizes range-wide.

**Conclusions:**

Substantial influences of geography, elevation and climate variables on genetic differentiation were also detected and may interact with the regional effects of anthropogenic climate change to force the loss of unique genetic lineages through continued population extirpations in the Great Basin and Sierra Nevada.

## Background

The impact of global climate change on species with wide geographic distributions is of particular interest given the greater likelihood that refugia from climate-mediated extirpations may exist for these taxa [1]. For widely dispersed alpine mammals that are already constrained by warming ambient air temperatures, the habitat characteristics that correlate with population persistence across mountain ranges will help define those areas where refugia may be found [2–5]. Although the complex topography and temporal variability of mountain ecosystems may offer refugia across relatively small spatial scales (kilometers), anthropogenic climate change threatens to test the limits of such microhabitat buffering [3, 6–9]. Therefore, in addition to habitat predictors of persistence, connectivity among habitat patches via gene flow may be critical to maintain genetic diversity and evolutionary potential within species [10–12]. Here we examine extant genetic variation within and among American pika (*Ochotona princeps*) populations sampled from diverse habitats across the western United States. We consider variation across geographic scales shaped by both historical (Pleistocene-era) and contemporary levels of connectivity.

The American pika has become a canary-in-the-coal-mine for the effects of anthropogenic climate change in montane habitats, as a result of extensive extirpations from warmer, lower elevation sites over the past two decades [13–17]. This small lagomorph is found in the mountainous regions of western North America where it lives primarily on talus slopes above timberline [18]. Talus (rocky slopes formed by freeze–thaw processes) provides thermal refuge for the pika, which has a relatively narrow thermal tolerance, and thus relies heavily on the stable thermal microclimate provided by the interstitial spaces of this rocky habitat [19, 20]. The American pika is one of only 30 *Ochotonidae* species worldwide. Most species are found in Asia and Eastern Europe with one North American congener in Alaska and Canada, *O. collaris* [18, 21].

Based upon analyses of Sanger sequenced mitochondrial (mtDNA) and nuclear DNA loci [22, 23], as well as allozyme [24], morphological [23] and behavioral data [23, 25, 26], Hafner and Smith [21] suggested collapsing the previously recognized 36 subspecies of *O. princeps* [18] into five subspecies. These designations are congruent with mitochondrial lineage designations [22] and correspond to distinct mountain ranges and provinces across western North America (i.e., Cascade and coastal ranges; Sierra Nevada and Great Basin; central Utah; Northern Rocky Mountains; and Southern Rocky Mountains). The origin of these lineages dates to 1.3 mya for the basal split between Cascade and Sierra Nevada lineages, and 0.8 mya for the subsequent divergence of Central Utah, Northern Rocky Mountain, and Southern Rocky Mountain lineages [23]. Repeated warming and cooling periods throughout the Pleistocene drove alternating range expansions and contractions. As the climate began warming at the end of the Pleistocene, *O. princeps* retreated upslope to higher elevations in the Sierra Nevada, Cascade and Rocky Mountains as well as in numerous smaller mountain ranges in the Great Basin physiographic province [23, 27, 28]. Over the ensuing 8000–10000 years, pika populations were lost from many mountain ranges in the Great Basin, particularly from low elevation ranges that lack sufficient talus habitat to provide refuge in a warming climate [29, 30]. Climate change in the twentieth and twenty-first centuries has further accelerated population declines and losses primarily from the Sierra Nevada lineage, *O. p. schisticeps* [17, 31–33]. This is especially true for the mountain ranges of the Great Basin, where resurveys of sites occupied in the mid-twentieth century revealed extensive extirpations by the early to mid-2000s [13, 15, 31].

In the few range-wide genetic studies that have been conducted for pikas, data suggest low levels of diversity within lineages [23, 24, 28]. However, within lineage population genetic data come from a few and mostly geographically distant populations characterized using a small number of traditional molecular markers (allozymes, mtDNA and nuclear introns and microsatellite loci) [22–24, 34]. As a result, detailed information on the amount and spatial structuring of variation within lineages is limited. A few studies have examined gene flow among populations separated by shorter distances of 0.5–10 km in both high elevation continuous or semi-continuous habitats and in marginal habitat or at the range periphery [35–38]. While these studies reveal significant population genetic structure at small spatial scales, patterns are not always consistent with an isolation-by-distance model, thereby strongly suggesting an interaction between habitat features and gene flow [39]. Because of naturally fragmented habitat and low dispersal capabilities, local extinction and colonization suggestive of a metapopulation dynamic has been proposed for this species at multiple spatial and temporal scales [28, 35, 40, 41].

Several recent studies have used more thorough genomic sampling to evaluate the influence of environmental variation and gene flow on fine scale patterns of genetic variation across populations at small geographic scales. Russello et al. [42] generated genotyping-by-sequencing (GBS) data to identify genomic regions responding to selection across four pika populations found along an elevational gradient within the coastal pika lineage in British Columbia. Genome-wide estimates of genetic diversity showed that the warmer, lower elevation populations had significant heterozygote deficiency suggestive of inbreeding and representing potential sink habitat [42]. In an additional study based on similar data, Waterhouse et al. [43] revealed directional gene flow from high to low elevation sites, which suggests that low elevation pika populations and any unique genetic variation they harbor may be lost in local extirpation events. In general, however, we largely lack an understanding of how genetic structure and diversity in pikas varies across ecoregions and habitats. Additional high throughput sequencing data for pika populations sampled across a continuum of spatial scales is likely to improve our understanding of patterns of population genetic structure, standing genetic variation, and their environmental correlates.

**Fig. 1:**
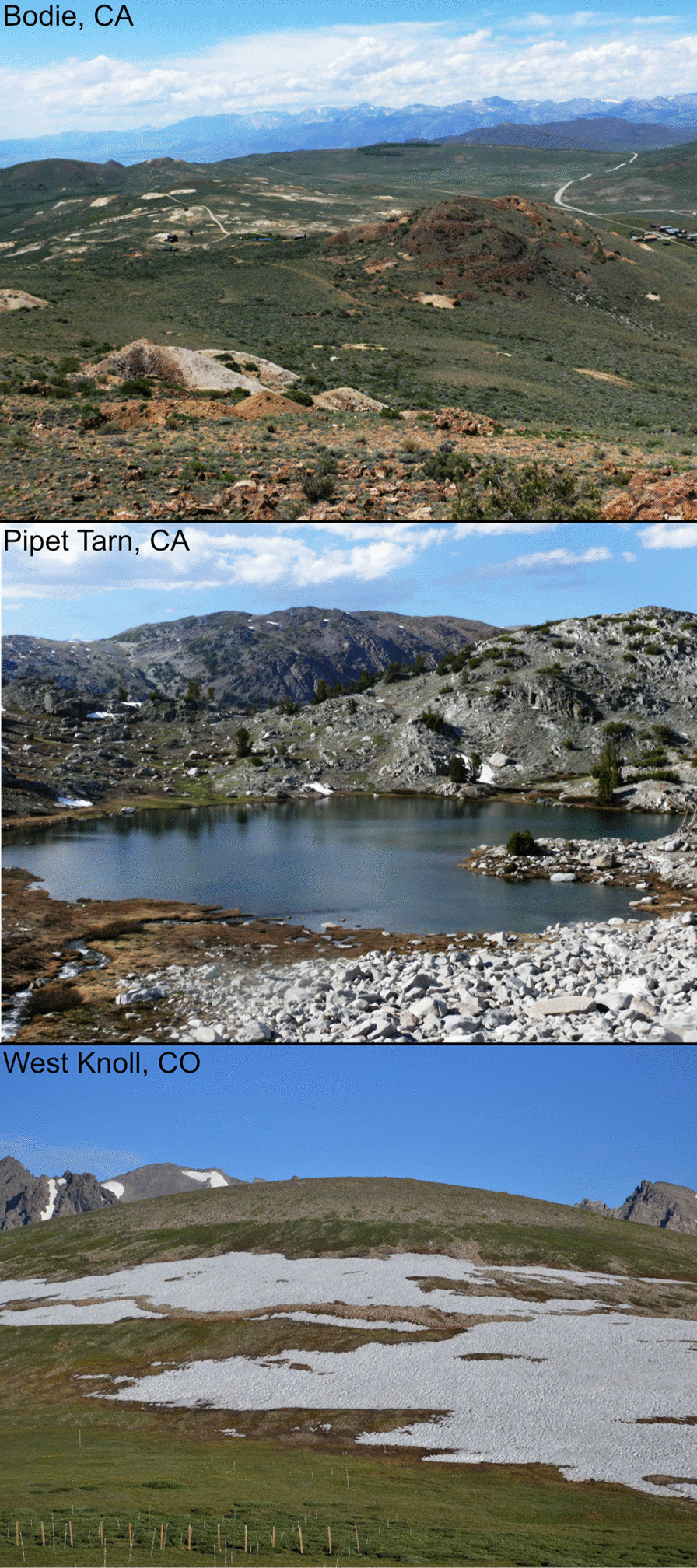
Photographs for three of the study areas from California and Colorado showing habitat diversity (Bodie Hills, Pipet Tarn, West Knoll). Photographs provide by authors KBK (Bodie and Pipet Tarn) and CR (West Knoll)

The populations sampled for this study represent three of the five *O. princeps* mitochondrial lineages as well as sampling locations that span the diversity of talus habitat found across the species’ range (Table 1; Fig. 1). We sampled two populations from the Sierra Nevada lineage, which comprises populations in the large Sierra Nevada range as well as the majority of smaller Great Basin mountain ranges. These populations are found in contrasting habitats separated by 38 km: one in high elevation continuous or semi-continuous talus habitat in the Sierra Nevada (3170 m; “Pipet Tarn”), and the other in lower elevation, highly fragmented habitat in the Bodie Hills (2500 m; “Bodie”), a lower elevation spur to the main Sierra Nevada range. Both of these populations have been the subject of earlier genetic studies assessing movement dynamics and gene flow under widely differing habitat spatial structures [35, 38, 44]. The lower elevation site has been the focus of extensive research on metapopulation dynamics in highly fragmented habitat [35, 40, 41, 45].

**Table 1:** Sample information for 11 pika populations used in this study which represent three subspecies (*O. p. princeps, O. p. schisticeps, and O. p. saxitilis*) and three of the five mtDNA lineages (Sierra Nevada, Northern and Southern Rocky Mountains)

Population	*N*	Elevation (m)	Latitude	Longitude	Sampling years	Subspecies
Bodie, CA	15	2500	38.211861	− 119.005037	2014–2015	*O. p. schisticeps*
Pipet Tarn, CA	20	3170	37.945285	− 119.28413	2014–2015	*O. p. schisticeps*
West Knoll, CO	21	3612	40.056753	− 105.59571	2013–2015	*O. p. saxatilis*
Emerald Lake, MT	21	2896	45.407061	− 110.93998	2011–2015	*O. p. princeps*
Swan Creek, MT	7	1829	45.374392	− 111.14408	2013	*O. p. princeps*
Overland Lake, NV (RM)	17	2281	40.458067	− 115.45524	1999–2000	*O. p. princeps*
Island Lake, NV (RM)	16	3245	40.616662	− 115.37916	1999–2000	*O. p. princeps*
Hidden Lake, NV (RM)	15	3051	40.746363	− 115.2845	1999–2000	*O. p. princeps*
Week’s Creek, NV (EH)	10	2825	40.928332	− 115.11273	1999–2000	*O. p. princeps*
Lizzie’s Basin, NV (EH)	10	2803	40.943973	− 115.11243	1999–2000	*O. p. princeps*
Smith Lake, NV (EH)	19	2882	41.034497	− 115.094786	1999–2000	*O. p. princeps*

**Fig. 2:**
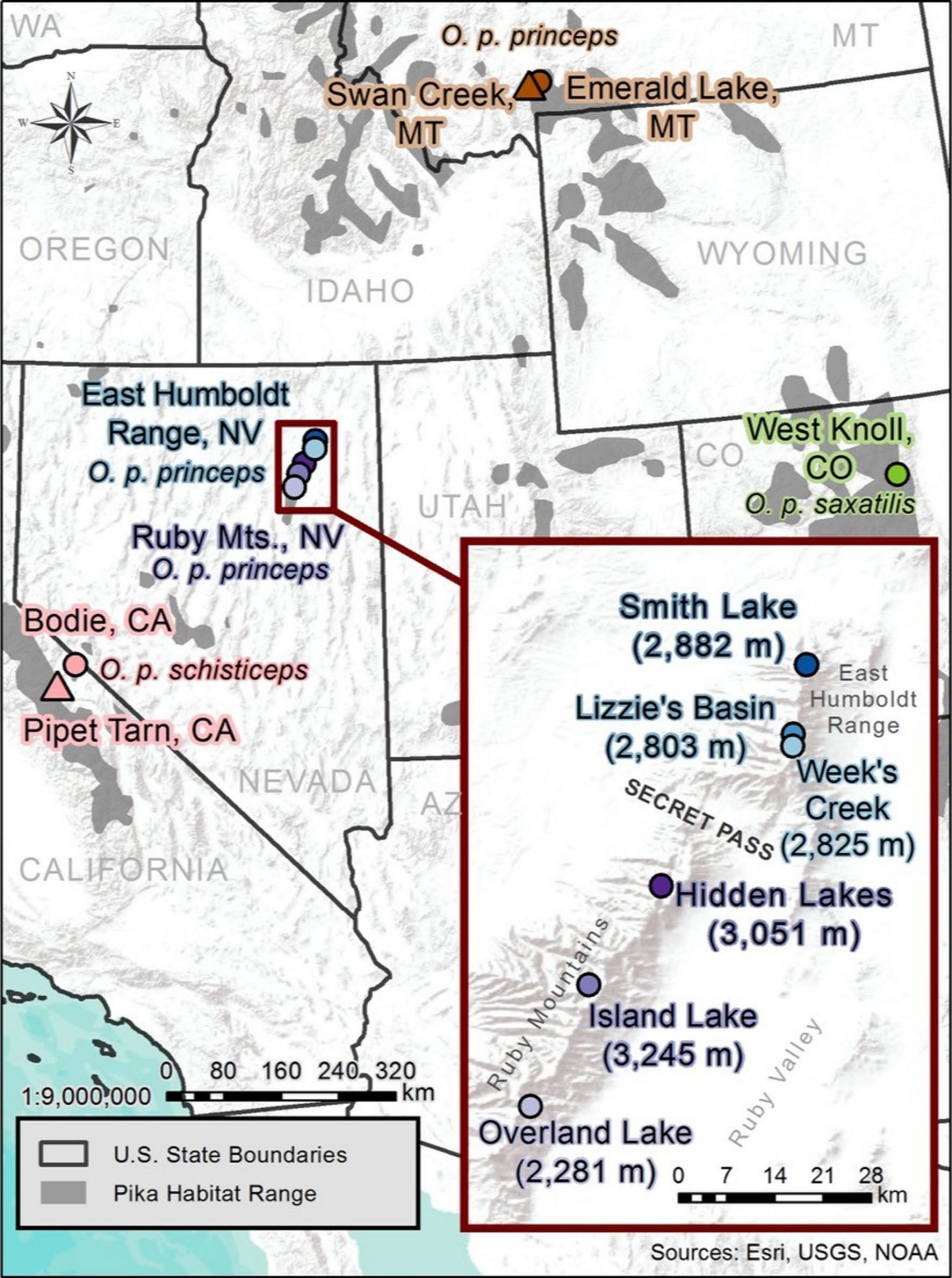
Map illustrating the 11 sampling locations of American pika (*Ochotona princeps*) populations distributed across the western United States. Shape and color correspond to region and sampling location and match precisely with the color and shapes used in subsequent figures. Populations representing the three sampled subspecies (*O. p. princeps*, *O. p. schisticeps*, and *O. p. saxatilis*) are labeled with text on the map. Figure created by author KBK

We also include populations from the Ruby Mountains and the East Humboldt Range in eastern Nevada, which are geographically adjacent and separated by a low elevation pass (~ 2800 m; Secret Pass; Fig. 2). Despite being located in the Great Basin, previous genetic work has shown that these populations represent the western most peripheral extent of the Northern Rocky Mountain mtDNA lineage [22]. The Great Basin contains several hundred, relatively small mountain ranges separated by wide low elevation desert basins, which serve as effective barriers to gene flow for small mammals [29, 46]. These mountain ranges are also strikingly linear and narrow, with a much smaller spatial extent than the Rocky Mountains and Sierra Nevada [29]. However, the Ruby Mountains and the East Humboldt Range constitute one of the largest contiguous montane habitats in the Great Basin (141 km^2^ above 2280 m) [47], with extensive talus that supports one of the largest pika populations remaining in this physiographic province. The remaining populations sampled for this study are from the Northern and Southern Rocky Mountains and occupy large contiguous talus slopes where pika ecology has been under study for decades [48–50].

Here, we expand on previous genetic studies of *O. princeps* by using a reduced representation GBS approach (ddRADseq) [51, 52]. We use these data to quantify patterns of population genetic variation across differing spatial scales: (1) among lineages; (2) among populations within lineages; and (3) at a finer scale within an isolated region of the Great Basin. Based upon previous studies we expected significant genetic divergence among lineages and strong evidence for isolation of the Great Basin populations. We predicted concordance between levels of genetic diversity and overall habitat area within mountain ranges and therefore expected lower levels of genetic variation in the peripheral and isolated populations of the Bodie Hills and the Ruby-East Humboldt Mountains. Finally, we expected the spatial genetic structure of populations to be jointly influenced by geography and environment, both across the range and across finer scales within the isolated Great Basin populations.

## Results

Reduced representation GBS libraries sequenced on two lanes of the Illumina HiSeq 4000 platform generated ~ 366 million total reads after initial filtering for potential contaminant reads, including those representing Illumina adaptors and PCR primers. After de-multiplexing by barcodes and matching reads to individual sample IDs, we retained an average of 1,909,370 reads (sd ± 394,102) per individual across 171 individuals for analyses. Assemblies run with bwa aligned 86,751,462 reads across all individuals (mean of 507,318 mapped reads per individual) to the *O. princeps* reference genome [56]. We identified 144,611 SNPs in the combined alignments of these individuals, but after filtering based on mapping quality, base quality, coverage and minor allele frequency, we retained a subset of 27,670 SNPs, with mean coverage depth per individual per locus of 6.2×, for downstream analyses (Fig. 2).

### Spatial genetic structure

**Fig. 3:**
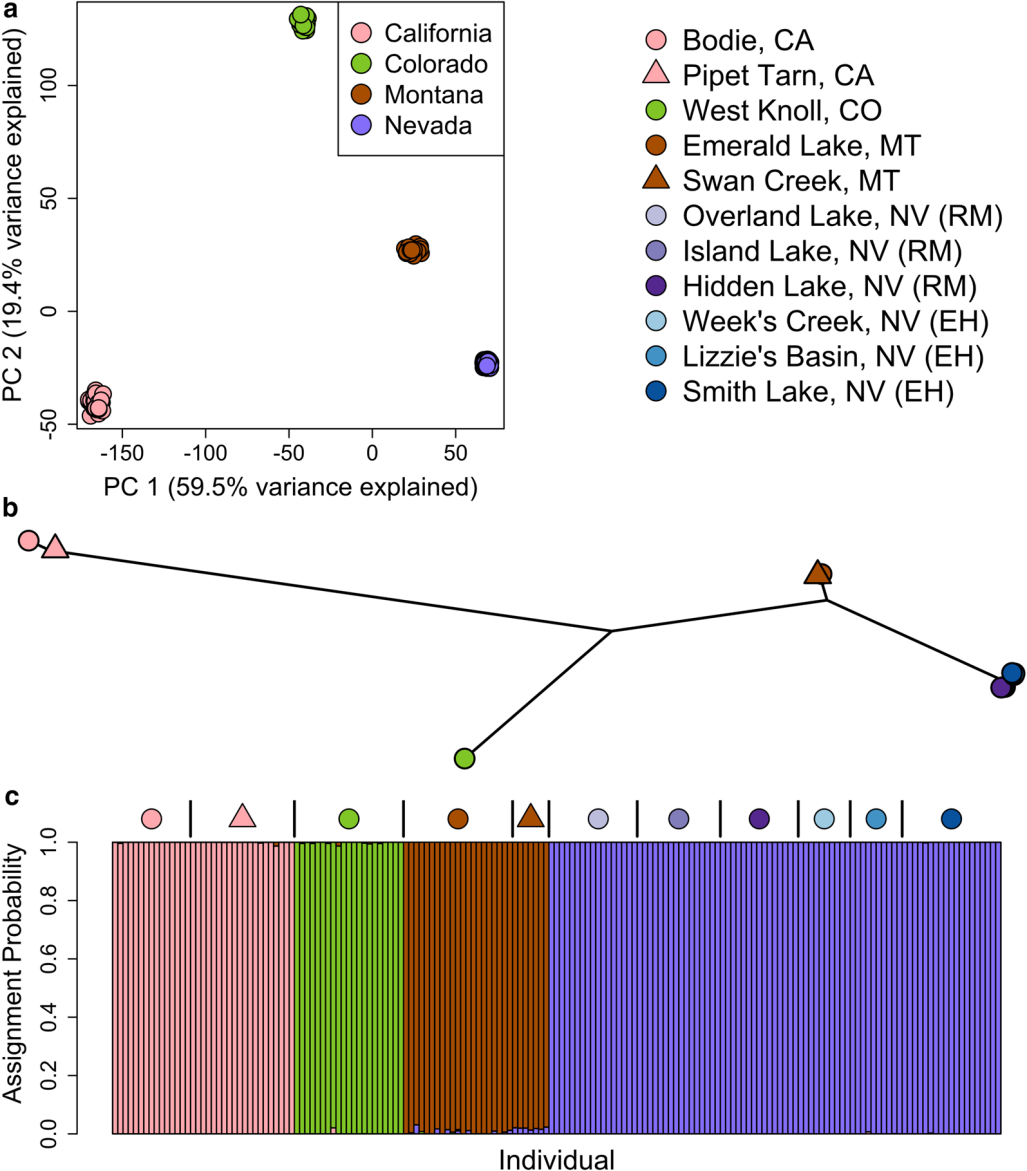
Population genetic structure of 11 populations of the American pika (*Ochotona princeps*) sampled from California (*O. p. schisticeps*), Colorado (*O. p. saxitilis*), Nevada (*O. p. princeps*) and Montana (*O. p. princeps*) based on 27,670 SNPs. **a** The first and second principal components (PCs) from the PCA are plotted for each individual (points were jittered to avoid overplotting). **b** An unrooted neighbor-joining tree analysis produced a tree topology reflecting strong evolutionary differentiation among pika subspecies. **c** A hierarchical Bayesian model (entropy) shows the assignment probability estimated for each individual for each of four genetic clusters (*k* = 4). Symbols are only used to delineate different populations within the same region and do not correspond to those found in Fig. 2. Figure created by author JPJ

We used a hierarchical Bayesian model (entropy) and PCA to characterize regional-scale (CA, MT, NV, and CO) genetic structure using the entire dataset of 171 individuals. entropy analyses supported four differentiated genetic clusters (*k* = 4) (Additional file 1: Table S1), and all individuals exhibited ancestry coefficients of close to 100% for one of the four (Fig. 3). Three of the clusters represented the subspecies, *O. p. princeps* (Montana samples; brown), *O. p. saxatilis* (Colorado samples; green) and *O. p. schisticeps* (eastern California samples; pink). The fourth cluster was comprised of *O. p. princeps* individuals from the Ruby-East Humboldt Mountains (Nevada samples; purple). PCA revealed patterns of population genetic structure consistent with the ancestry estimates from entropy (Fig. 3). The first two principal components (PCs) accounted for 59.5 and 19.4% of the variation in the genotype probabilities, respectively, and revealed significant differentiation among all three subspecies (*O. p. princeps, O. p. schisticeps, O. p. saxitli*s) as well as *O. p. princeps* from Nevada (Fig. 3). The first PC separated *O. p. princeps* individuals (Montana and Nevada populations), while PC2 identified a distinct break between individuals sampled from the southwestern region of the species’ range (California and Nevada individuals, *O. p. schisticeps* and *O. p. princeps*, respectively) and the northeast region (Colorado and Montana; *O. p. saxitilis* and *O. p. princeps*, respectively) (Fig. 3).

Estimates of Nei’s *D* indicated the presence of hierarchical genetic differentiation within and among subspecies and geographic regions (Fig. 3b and Additional file 2: Fig. S1). The Sierra Nevada lineage (*O. p. schisticeps*) was highly differentiated from both the Northern Rockies lineage (*O. p. princeps*; Nei’s *D* range: 0.400–0.503) and the Southern Rockies lineage (*O. p. saxatilis*; Nei’s *D* range: 0.376–0.390). The Southern Rockies lineage was also well differentiated from the Northern Rockies lineage (Nei’s *D* range: 0.214–308). Within *O. p. princeps*, Great Basin populations exhibited modest differentiation from Montana populations (Nei’s *D* range: 0.111–0.116). Finally, genetic distances between populations from the Ruby Mountains and the East Humboldt Range were relatively small (Nei’s *D* range: 0.011–0.012).

**Fig. 4:**
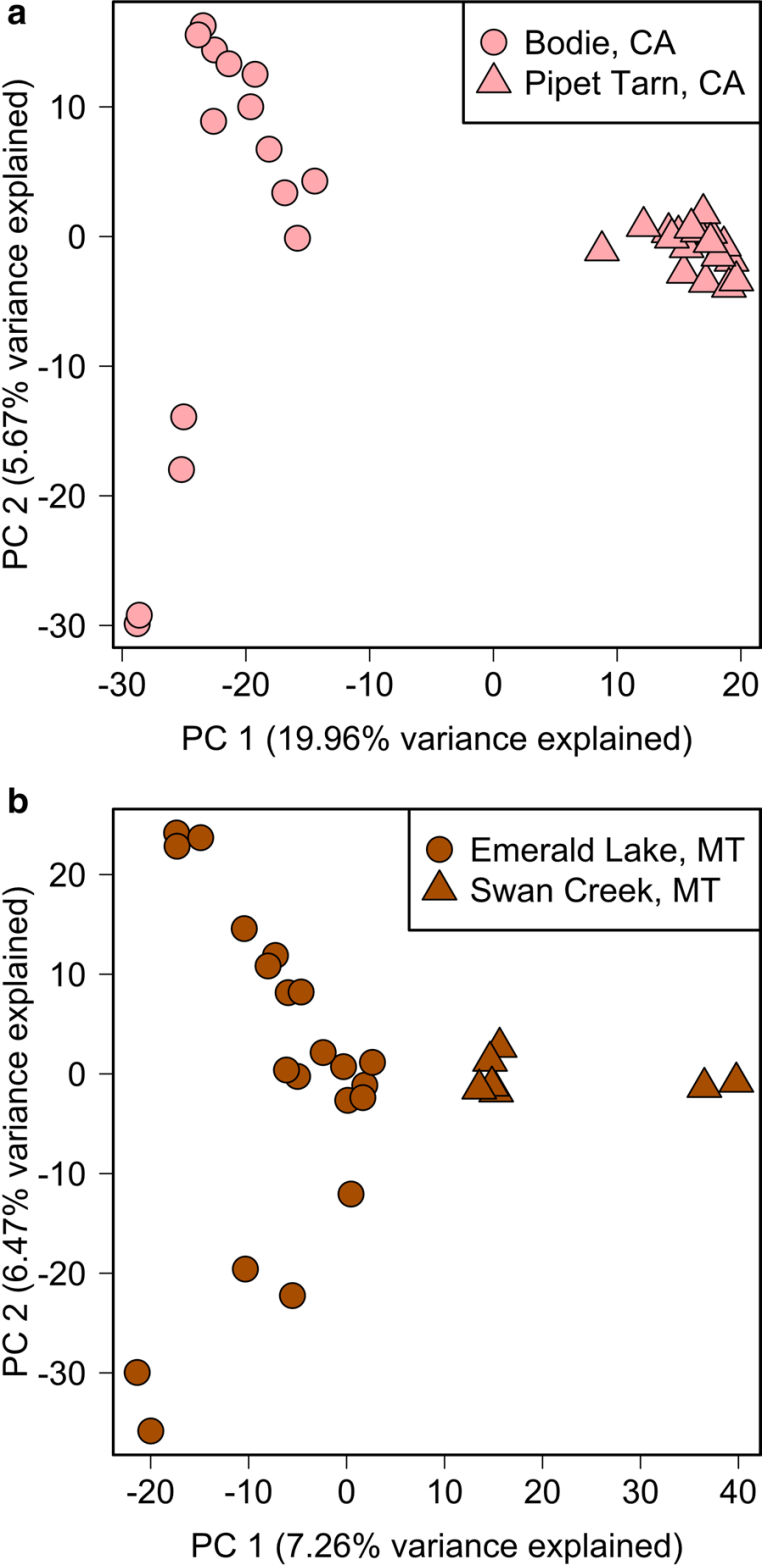
Principal components analysis is consistent with population genetic structure for American pika (*Ochotona princeps*) populations from **a** eastern California and **b** Montana. Symbols are only used to delineate different populations within the same region and do not correspond to those found in Fig. 2. Figure created by author JPJ

**Fig. 5:**
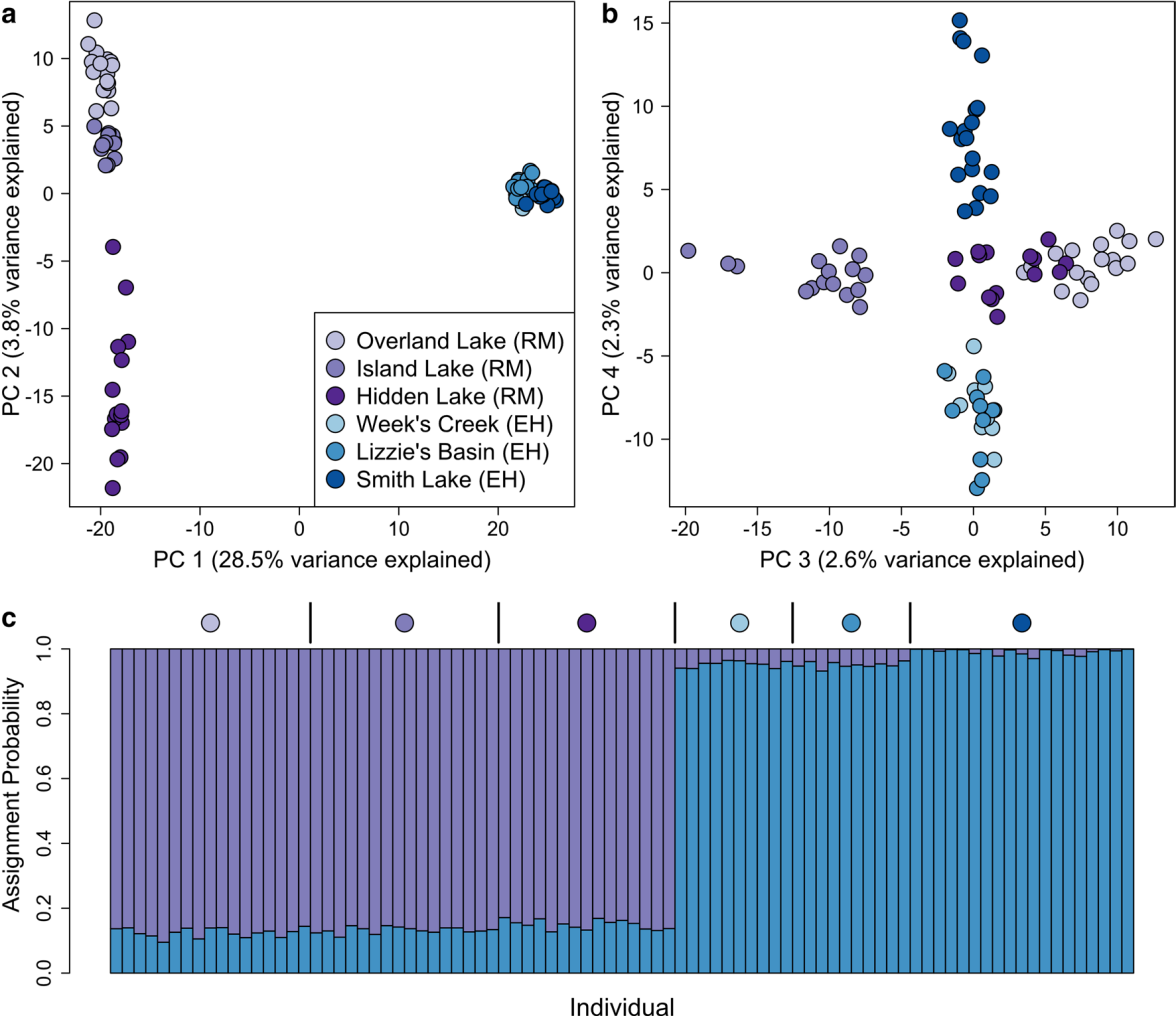
Population genetic structure of six populations of the American pika (*Ochotona princeps*) from the Ruby Mountains (RM) and the East Humboldt range (EH) in eastern Nevada. The **a** first, second, **b** third, and fourth principal components (PCs) from the PCA are plotted for each individual. **c** A hierarchical Bayesian model (entropy) shows the assignment probability estimated for each individual for each of two genetic clusters (*k* = 2). Symbols are used only to delineate different populations within the same region and do not correspond to those found in Fig. 2. Figure created by author JPJ

Despite low overall genetic divergence among populations within each lineage or region, our analyses consistently revealed evidence for fine-scale spatial structure within them. Based on PCA, clear differentiation was observed within lineages where the sampled populations were separated by large geographic distances, including the pair separated by ~ 18 km within the Northern Rockies (Emerald Lake and Swan Creek) and the pair separated by ~ 38 km within the Sierra Nevada (Bodie and Pipet Tarn) (Fig. 4). For the six Great Basin populations sampled within the Ruby-East Humboldt Mountains (separated by a maximum distance of 70 km) fine-scale spatial structure was particularly striking. The first two PCs explained 32.3% of the variance and strongly differentiated between populations separated by the low elevation Secret Pass within this otherwise continuous mountain range (Fig. 5a). The Island Lake population was separated along PC3 axis from the other two populations in the Ruby portion of the range, Overland Lake and Hidden Lake, despite its central location within the range. Smith Lake was separated from Lizzie’s Basin and Week’s Creek in the East Humboldt portion of the range along the PC4 axis (Fig. 5b). The entropy model based on *k* = 2 (Fig. 5c) was strongly supported, but the *k* = 6 model had similar support and reflected clear fine scale spatial structure analogous to PC3 and PC4 (Fig. 5b, Additional file 1: Table S2).

### Geographic and environmental predictors of spatial genetic structure

**Fig. 6:**
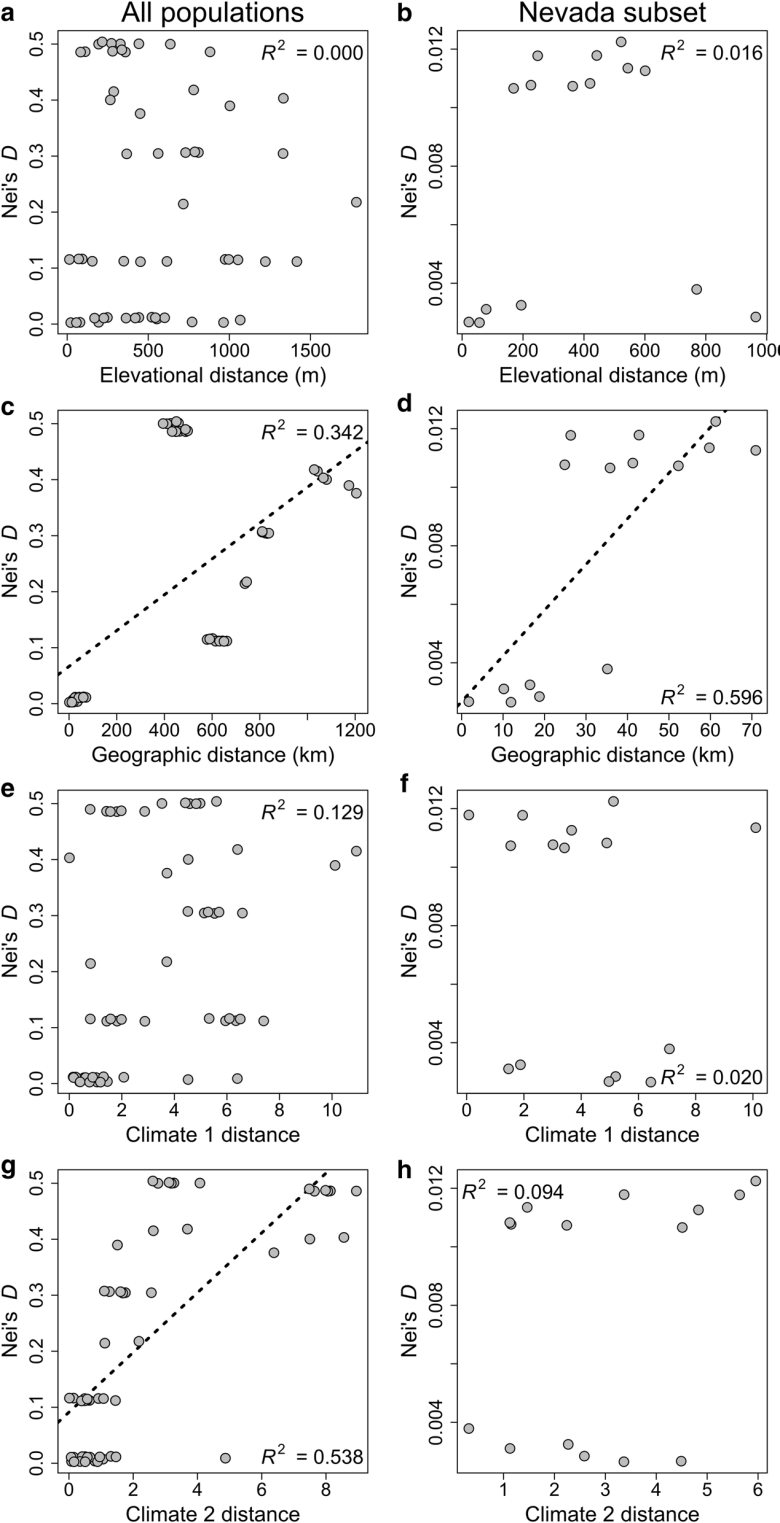
The relationship between Nei’s *D* and elevational distance (m), haversine geographic distance (km), climate 1 and climate 2 predictor variables (19 climatic variables downloaded from https://www.worldclim.org; see supplementary tables for specific variable identities). Panels **a**, **c**, **e**, and **g** represent all sampled pika populations, and panels **b**, **d**, **f**, and **h** represent the subset of six Nevada populations located along an elevational gradient between the Ruby Mountains and East Humboldt Range. Figure created by author JPJ

Across all study populations, ~ 80% of the climatic variation was explained by the first two PCs. The first climate PC (hereafter Climate 1) had strong loadings for variables related to temperature, whereas precipitation and seasonality variables loaded more heavily on the second climate PC (hereafter Climate 2) (Additional file 1: Table S3). Of the four predictor variables, only geographic distance and Climate 1 distance were strongly associated with one another across all populations (*R*^2^ = 0.344; *P* = 0.010; Additional file 3: Fig. S2). Nei’s *D* was strongly predicted by Climate 2 distance (*R*^2^ = 0.538; *P* = 0.009) and geographic distance (*R*^2^ = 0.342; *P* = 0.009), but was not influenced by Climate 1 distance (*R*^2^ = 0.129; *P* = 0.079) or elevational distance (*R*^2^ = 0.000; *P* = 0.960) (Table 2; Fig. 6). A model with both Climate 2 distance and geographic distance explained 67.8% of the variation in Nei’s *D* and had comparable explanatory power relative to models containing more parameters (Table 2).

**Table 2:** Comparison of MRM tests predicting Nei’s genetic distance (*D*) among all 11 populations

Model	*R*^2^	*P*
Elevation + Geography + Climate 1 + Climate 2	0.716	0.005
Geography + Climate 1 + Climate 2	0.708	0.003
Elevation + Geography + Climate 2	0.695	0.002
Geography + Climate 2	0.678	0.002
Elevation + Climate 1 + Climate 2	0.673	0.001
Climate 1 + Climate 2	0.673	0.001
Elevation + Climate 2	0.538	0.005
Climate 2	0.538	0.002
Elevation + Geography + Climate 1	0.385	0.057
Elevation + Geography	0.384	0.038
Geography + Climate 1	0.342	0.026
Geography	0.342	0.009
Elevation + Climate 1	0.130	0.184
Climate 1	0.129	0.079
Elevation	0.000	0.960

The two climate variables correspond to the first two principal components from a PCA on 19 climate variables from BioClim, which explained 46.9 and 33.8 percent of the variance in the data, respectively (see Table S4 for loadings and Fig. S2 for relationships among predictor variables)

For the Great Basin subset of populations, ~ 91% of the variation in climate among sites was explained by the first two PCs. As in the full dataset above, the first two PCs had strong loadings for variables related to temperature and precipitation, respectively, but seasonality variables loaded more strongly on PC1 for the Nevadan subset (Additional file 1: Table S4). Elevational and geographic distance were correlated with one another for the subset of Nevadan populations (*R*^2^ = 0.250; *P* = 0.046), but neither climate variable was strongly coupled with any other predictor (Additional file 3: Fig. S2). Nei’s *D* among populations in the Ruby Mountains and East Humboldt Range was strongly affected by geographic distance (*R*^2^ = 0.596; *P* = 0.024), but was not associated with Climate 1 distance (*R*^2^ = 0.020; *P* = 0.399), Climate 2 distance (*R*^2^ = 0.094; *P* = 0.130), or elevational distance (*R*^2^ = 0.016; *P* = 0.284) (Table 3; Fig. 6). Alternative models including geographic distance plus additional predictors did have improved explanatory value, but the gains were fairly modest relative to the more parsimonious model including only geographic distance (Table 3).

**Table 3:** Comparison of multiple regression on distance matrices (MRM) predicting Nei’s genetic distance (*D*) among the six Nevadan populations

Model	*R*^2^	*P*
Elevation + Geography + Climate 1 + Climate 2	0.727	0.024
Elevation + Geography + Climate 1	0.722	0.024
Elevation + Geography + Climate 2	0.695	0.034
Elevation + Geography	0.686	0.025
Geography + Climate 1 + Climate 2	0.685	0.013
Geography + Climate 1	0.670	0.017
Geography + Climate 2	0.628	0.021
Geography	0.596	0.024
Elevation + Climate 1 + Climate 2	0.149	0.243
Elevation + Climate 2	0.126	0.144
Climate 1 + Climate 2	0.102	0.227
Climate 2	0.094	0.130
Elevation + Climate 1	0.055	0.242
Climate 1	0.020	0.399
Elevation	0.016	0.284

The two climate variables correspond to the first two principal components from a PCA on 19 climate variables from BioClim, which explained 60.0 and 31.0 percent of the variance in the data, respectively (see Additional file 1: Table S5 for loadings and Additional file 3: Fig. S2 for relationships among predictor variables)

### Levels of genetic diversity

**Fig. 7:**
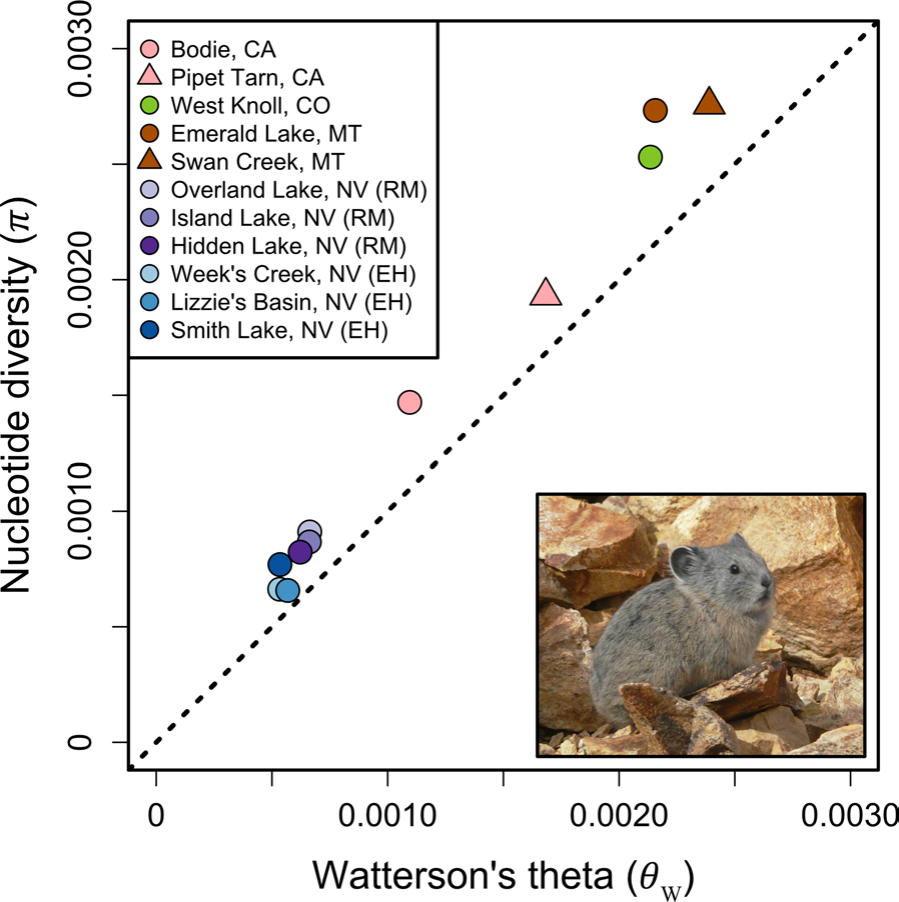
Estimates of Watterson’s theta (θ_W_) and nucleotide diversity (π) are displayed for all eleven sites. Symbols are used only to delineate different populations within the same region and do not correspond to those found in Fig. 2. Insert: a pika at Bodie Hills, CA. Photo provided by author KBK. Figure created by author JPJ

Estimates of nucleotide diversity (π) [77] and Watterson’s theta (θ_W_) [78] were highly variable across the sampled localities (π range: 0.0006–0.0027; θ_W_ range: 0.0005–0.0023; Fig. 7; Additional file 1: Table S5). Populations from Montana and Colorado had the highest levels of standing variation (π range: 0.0025–0.0027; θ_W_ range: 0.0021–0.0023), while those sampled in the Ruby-Humboldt ranges had by far the lowest (π range: 0.0007–0.0009; θ_W_ range: 0.0005–0.0007) (Fig. 7; Additional file 1: Table S5). All populations had positive estimates of Tajima’s *D* (consistent with recent population contraction), with the largest estimate found in Bodie, CA (Tajima’s *D* = 0.811) and the lowest estimate in Lizzie’s Basin, NV (Tajima’s *D* = 0.240) (Additional file 1: Table S5).

## Discussion

For many North American taxa, including the pika, persistence in Pleistocene-era refugia enabled survival across repeated glacial cycles and led to strongly divergent populations or lineages [22, 23, 87]. Our results support earlier range wide analyses conducted by Galbreath et al. [22, 23], which show clear genetic differentiation among the three pika mtDNA lineages sampled here—Northern Rockies, Southern Rockies and Sierra Nevada—consistent with glacial cycles and periods of isolation during the Pleistocene. The Galbreath et al. [22] phylogeny based upon mtDNA cytochrome *b* and D-loop sequence data placed the populations in the Ruby-Humboldt Mountains of Nevada into a clade with the other southern most populations of the Northern Rocky Mountain lineage (*O. p. princeps*). However, additional sequence data from nuclear introns did not further clarify relationships among the Ruby-East Humboldt populations and other populations in the Northern Rocky Mountain clade despite the geographic isolation of this mountain range [23]. In contrast, increased genomic sampling in our data provides clear evidence of differentiation of the Ruby-East Humboldt populations from other populations in the Northern Rocky Mountain clade. Bayesian clustering analyses, PCA, and pairwise estimates of genetic distance from this study reveal the distinctiveness of the pikas in the Ruby-East Humboldt Mountains.

### Spatial genetic structure and its predictors

While geographic distance predicted genetic distance among all populations, a composite climate distance (Climate 2; precipitation and seasonality variables) was more strongly associated with genetic distance (Table 2, Additional file 1: Table S3; Fig. 6). Importantly, geographic and Climate 2 distance were not correlated with one another across all populations (Additional file 3: Fig. S2), suggesting that our results are consistent with both isolation-by-distance and isolation-by-environment [88] and that the evolutionary history of American pikas has been shaped in part by adaptation to climatic variability across the species range. Indeed, the relative importance of the interaction between temperature stress and the seasonality [89], location [90], and form of precipitation (i.e. rain, snowpack, moisture) [33] are increasingly being recognized as important determinants of pika distribution and persistence under a changing climate. Unfortunately, our sequencing dataset—and RADseq/GBS datasets in general—has limited utility for identifying genomic regions potentially associated with local adaptation because the decay of linkage disequilibrium typically occurs at a scale much smaller than typical marker densities ([91–93]; but see [94, 95]). Marker density for our dataset was roughly one locus per 80,000 bp, suggesting that more thorough sequencing will be needed to effectively survey the genome.

In contrast, genetic distance at smaller spatial scales within the Ruby Mountains and East Humboldt Range was overwhelmingly associated with geographic distance (Table 3; Fig. 6). A lack of evidence supporting isolation-by-environment for the Great Basin populations in the Ruby-East Humboldt range is not surprising, as they experience similar climatic conditions, diverged relatively recently (Fig. 3b and Additional file 2: Fig. S1), and were potentially connected during the Last Glacial Maximum when the upper reaches of the Ruby Mountains and the East Humboldt Range were glaciated [96]. Isolation-by-distance has been previously reported for Great Basin pikas [97] and has also been documented in other Great Basin alpine organisms, including marmots [98] and forbs [99].

### Variation in genetic diversity across lineages and populations

Genetic diversity was an order of magnitude lower in the Ruby-East Humboldt range populations compared to all other populations sampled (Table 3), including the Bodie Hills population, which is found in highly fragmented habitat surrounded by desert-shrub vegetation and isolated from the main Sierra Nevada range. Interestingly, the nucleotide diversity estimates in Ruby-East Humboldt pika populations were similar to those generally found for arctic and subarctic alpine mammals (ddRADseq data: collared pika, *O. collaris*, π = 0.00011–0.00034; hoary marmot, *Marmota caligata*, π = 0.00009–0.00063; singing vole, *Microtus miurus*, π = 0.00032–0.00089; brown lemming, *Lemmus trimucronatus*, π = 0.00079–0.00108; arctic ground squirrel, *Urocitellus parryii*, π = 0.00043–0.00069; [100]). The low levels of genetic variation in these arctic species are likely due to the influences of limited glacial refugia during the Pleistocene, as dispersal ability did not explain the observed genetic patterns [100].

In a general sense, the levels of genetic diversity within the pika populations sampled for this study were related to habitat size, insofar as populations from the larger mountain ranges had higher diversity levels (Fig. 7; Additional file 1: Table S5). Although the majority of our sampled populations are located in high elevation habitat with continuous to semi-continuous talus, such habitat characteristics in and of themselves did not ameliorate the loss of genetic variation in the Ruby-East Humboldt populations. In contrast, although talus habitat is limited to widely spaced rocky outcrops across the Bodie Hills plateau with very few sites currently occupied [101], the Bodie Hills population sampled for this study inhabits ~ 100 talus patches formed from ore dumps associated with hard rock gold mining in the later nineteenth century [35, 102, 103]. Genetic diversity at this site was similar to the levels observed in the Rocky Mountains and Sierra Nevada. The difference in levels of genetic diversity found between the Ruby-East Humboldt range and the Bodie Hills, especially given the differences in habitat, suggests potential roles for both the spatial extent of habitat and configuration of local habitat patches in influencing rates of gene flow and thus the maintenance of genetic variation [35].

### Metapopulation dynamics, local habitat spatial structure and maintenance of genetic diversity

An extinction and colonization dynamic among semi-independent subpopulations underpins metapopulation theory [104] and predicts reductions in overall genetic diversity in metapopulations over time for scenarios of limited source populations, low recolonization rates, and genetic drift [105–108]. There are numerous empirical examples from a diverse set of taxa that associate lower genetic diversity with a metapopulation dynamic (e.g., hyrax, *Heterohyrax brucei* and *Procavia johnstoni* [109]; moths, *Aglaope infausta* [110]; trout, *Oncorhynchus clarkii henshawi* [111]; caddisflys, *Allogamus uncatus* [112]; treefrogs, *Hyla wrightorum* [113]).

The fragmented nature of pika habitat, whether at small spatial scales as in the Bodie Hills or larger habitat patches in mountain ranges such as in the Ruby-East Humboldt, Sierra Nevada or Rocky Mountains, suggests pikas across their range may experience metapopulation dynamics albeit at very different temporal and spatial scales, which in turn may result in differing genetic signals. Metapopulation dynamics may therefore partially explain the low genetic diversity observed in pika populations found in the Ruby-East Humboldt range.

The basin and range topography of the Great Basin dates to approximately 30 million years ago and pikas are thought to have colonized these interior basin mountain ranges more recently during the Pleistocene [29]. As the climate warmed, pikas disappeared from most of the Great Basin mountains ranges and are currently found only in the largest ranges with the highest elevational extent [29]. The genetic data from this study suggest that the pika populations in the Ruby-East Humboldt range have long been isolated from other populations in their own lineage. The narrow linear configuration of the Ruby-East Humboldt mountain range limits the amount and spatial extent of talus and may thus constrain both dispersal distance and direction. Such limitations may increase population genetic structure and reduce colonization potential of extirpated talus patches. Although the Bodie Hills plateau was likely colonized by pikas from the main Sierra Nevada also during the Pleistocene, this high elevation spur of the main range has remained connected to the Sierra Nevada and thus ongoing gene flow was at least possible. However, the population sampled for this study is one of the very few extant populations remaining in the Bodie Hills. As a result, the genetic diversity found within the Bodie Hills population might seem anomalous given that it is an isolated metapopulation in highly fragmented habitat [35, 41, 45]. Earlier genetic work using DNA minisatellite markers showed that average heterozygosity for the Bodie Hills population was similar to the Pipet Tarn population inhabiting the larger and contiguous habitat of the main Sierra Nevada [35]. One explanation for the relatively high genetic diversity at Bodie (Table 3), despite the effects of genetic drift, involves the large number of small habitat patches at this site as well as a relatively recent founding event, which is estimated to have occurred sometime during the late nineteenth century [103, 114, 115]. If extinction probabilities are uncorrelated among these patches, then Bodie can act as a highly persistent metapopulation of small local populations that drift toward fixation for different alleles, thereby maintaining genetic diversity at the metapopulation level similar to that of a large un-subdivided population [115]. Furthermore, dispersal among extant patches has been very fluid in this population, driven by juveniles leaving small patches (when territories are unavailable) and having multiple habitat patches within dispersal distance [35, 38]. Therefore, ongoing within-population dispersal may have acted to spread and maintain genetic variation among patches in between extinction events [35].

The role of spatial configuration of talus patches, in contrast to overall habitat size within mountain ranges, has not received as much attention as other potential correlates of extirpation or persistence probabilities for pika populations (but see [116]). Examples from the literature, for metapopulations that have retained genetic variation, suggest that ongoing dispersal and gene flow among extant subpopulations, in between extinction events, can in some cases be critical for maintenance of genetic diversity (e.g., natterjack toad, *Bufo calamita* [117]; black-tailed prairie dogs, *Cynomys ludovicianus* [118]; the treacle-mustard, wormseed wallflower *Erysimum cheiranthoides* [119]; Galapagos warbler finches (*Certhidea*) [120]: cichlid fishes, *Eretmodus cyanostictus, Variabilichromis moorii* and *Tropheus moorii* [121]). Levels of genetic diversity within metapopulations will ultimately depend upon the number of local populations, extirpation-colonization rates and gene flow among local extant populations [105, 113, 114, 122].

### Implications for pika persistence

Our results agree with previous analyses suggesting vulnerability of Great Basin pika populations [13–15, 31, 32]. Pika populations in the Great Basin ecoregion represent two mtDNA lineages including the Sierra Nevada and Northern Rocky Mountains. The topography of the Great Basin effectively isolates populations within these ranges and while extirpations have been occurring since the end of the Pleistocene, they have accelerated over the late twentieth and early twenty-first centuries [13–15, 29]. Few of the extant pika populations in the Great Basin have been characterized genetically, but the distinctness of the populations in the Ruby-East Humboldt Mountains suggests these ranges may harbor unique genetic variation. The accelerated rate of pika population losses in recent decades in this ecoregion could certainly be associated with range isolation, mountain range size, as well as mean elevation and a warming climate [13, 15, 46]. This is especially concerning considering that the most evolutionarily distinct lineage sampled in this study (*O. p. schisticeps*; Fig. 2b) is also the lineage that occurs in both eastern California and the Great Basin [22, 23]. These two regions, which have produced most of the evidence for climate change-induced pika population extirpations [17, 33, 123, 124], are also predicted to lose more of the currently suitable pika habitat during this century [16, 22, 125].

Habitat fragmentation together with increasing temperatures and expansion of invasive species are reducing the habitable landscape for diverse alpine taxa including alpine vascular flora [126, 127], mammals [12, 128–130], and aquatic invertebrates [131, 132]. The Grinnell resurvey project in Yosemite National Park showed an upslope contraction of lower elevational limits for half of 28 small mammal species surveyed, consistent with the ~ 3 °C increase in minimum temperatures observed over the past century [128]. Despite the maintenance of genetic variation in some of the populations sampled for this study, Tajima’s *D* estimates indicate that all have suffered recent population contractions, with the Bodie Hills population showing the greatest contraction (Additional file 1: Table S5). Earlier work across California, which included the pika population at the Bodie Hills site, revealed that both temperature and habitat area influenced pika occupancy [17, 38]. There has also been a concomitant reduction in allelic diversity and effective population size in the Bodie Hills population over the past 25 years [35, 38].

For the American pika, climate change is likely to reduce the already low rates of gene flow among local populations [44, 133] through increased mortality and reduced dispersal [116], thereby intensifying the impacts of genetic drift on standing genetic variation. The majority of pika populations sampled had levels of nucleotide diversity (Fig. 7; Additional file 1: Table S5) below the average estimate for a wide range of taxa (green plants, animals, fungi and Chromalveolates; mean = 0.0065, π = 0.0005–0.05; [134]). However, mammalian species in general tend to have lower levels of diversity, compared to the other taxa, where diversity is likely to co-vary with geographic range and life history [100, 134]. The very low genetic diversity of the Ruby-East Humboldt populations raises concerns about the effects of genetic drift on genetic variation in isolated populations and highlights the need for more extensive sampling of this and other insular ranges.

## Conclusions

Expanding the genetic characterization of population level genetic diversity will be necessary to define fully the genetic variation within pika lineages and how this diversity is partitioned across the landscape. Our results suggest that the natural fragmentation inherent to rugged, mountainous terrain may have disproportionate effects [135] on pikas and other small alpine mammals by constraining connectivity critical for maintaining local demographic stability [41]. The patterns of climate-associated, range-wide differentiation observed in this study, along with the significant role of geography at finer scales, reinforces the need to tease apart the specific climatic or non-climatic factors that can shape genetic variation across small to moderate spatial scales within each mountain system. Indeed, while these findings provide important context regarding the effects of geography and climate on the amount and distribution of extant genetic diversity, our understanding of the potential adaptive diversity that these genetic data represent in pikas is unknown. Furthermore, while adaptive genetic diversity is likely to play a role in persistence, recent work has emphasized the relevance of landscape context at the ecoregional scale, not genetic lineage, in structuring intraspecific variation in response to climate change [136]. Given these insights, genomic analyses, like those conducted here, at within-mountain range scales, may be most useful for identifying those portions of the pika’s range that contain talus of sufficient quality, quantity and spatial configuration to support maintenance of species-wide genetic diversity.

## Methods

### DNA resources

All individual pikas included in this study were live-trapped and released at point of capture after processing. No animals were euthanized for this study. The majority of tissue samples were collected over the last ten years as part of long-term monitoring efforts or recent live-trapping studies (Table 1). Additional DNA samples curated from earlier studies were obtained for a subset of sites (Ruby Mountains [Overland Lake, Island Lake, Hidden Lake] and East Humboldt Range [Week’s Creek, Lizzie’s Basin and Smith Lake]; Montana sites, Emerald Lake and Swan Creek). Samples were collected from 11 distinct populations, which represent three subspecies (*O. p. princeps, O. p. schisticeps, and O. p. saxitilis*) and three of the five mtDNA lineages (Sierra Nevada, Northern and Southern Rocky Mountains) (Table 1; Fig. 2).

Live-trapping for pikas from the Bodie and Pipet Tarn sites was conducted as follows. Pikas were trapped using Tomahawk live traps (16″ × 6″ × 6″) baited with native alpine vegetation and covered with rocks to prevent exposure to weather and predators [35, 37]. Traps were set predawn and checked within 2 h. Trapped individuals were treated with an inhalant anesthetic (isoflurane) before a small sample of ear tissue (< 50 mg) was collected and placed in cryovials and stored in liquid nitrogen. Animals were released at the point of capture after 15 min of recovery. We followed the guidelines of the American Society of Mammalogists for live animal research [53]. Permission for live capture and release of pikas was approved by Institutional Animal Care and Use Committees (IACUC) at the University of Nevada Reno and University of Colorado, Boulder. Trapping permits were obtained from California Department of Parks and Recreation; California Department of Fish and Wildlife; Colorado Parks and Wildlife; Montana Fish, Wildlife and Parks; Nevada Department of Wildlife, and US Department of Agriculture Forest Service.

### Illumina sequencing of restriction fragment libraries

DNA was extracted from tissue samples representing 35 *O. p. schisticeps*, 21 *O. p. saxitilis*, and 115 *O. p. princeps* individuals (Fig. 2; Table 1) using a Qiagen DNeasy Blood and Tissue Kit and quantified on a QIAxpert UV/VIS spectrophotometer (Qiagen, Inc., Valencia, CA, USA). Reduced representation libraries were prepared for Illumina sequencing following a genotyping-by-sequencing (GBS) protocol [51] analogous to ddRADseq [52]. Genomic DNA was digested with two restriction endonucleases (*Eco*RI and *Mse*I) and double-stranded adaptor oligonucleotides were ligated to the digested fragments. Adaptor ligation occurred simultaneously with restriction digestion using adaptor sequences that incorporated unique 8–10 base pair (bp) barcodes for each individual, as well as the priming sites for Illumina sequencing. Ligated fragments were PCR amplified using a high-fidelity proofreading polymerase, uniquely barcoded products were pooled into a single library and then size selected for fragments ranging from 350 to 425 bp in length using a Blue Pippin unit (Sage Science, Beverly, MA, USA). Single end 150 bp reads were generated with two lanes of sequencing on an Illumina HiSeq 4000 at the University of Texas Genomic Sequencing and Analysis Facility (Austin, TX, USA).

### DNA sequence assembly, and variant calling

We first filtered out low-quality reads, those representing common bacterial contaminants, and those associated with Illumina oligos using bowtie_db2 [54] and the tapioca pipeline (https://github.com/ncgr/tapioca). Individual bar codes and the adjacent six bases corresponding to the *Eco*RI cut sites were then trimmed from each read, and individual identifiers were incorporated into separate fastq files for each individual (available at Dryad; [55]). We aligned all reads to the draft *O. princeps* genome (NCBI identifier: GCF_000292845.1; OchPri3.) [56] using the aln and samse algorithms of bwa v0.7.8 [57] with an edit distance of four and otherwise default parameter settings.

We used the mpileup command in samtools v0.1.19 [58] to merge the .bam alignment files of all individuals into .bcf formatted files. We subsequently used bcftools v0.1.19 [58]) to identify bi-allelic single nucleotide polymorphisms (SNPs) and to calculate genotype likelihoods (-e option). We used the default scaled substitution mutation rate, a full prior setting (-P option) and called SNPs if P(ref|D) < 0.01 (vcftools v0.1.14) [59] was used to perform additional quality filtering. We retained SNPs in our final data set when 92% of the individuals had at least one read at the locus. We excluded variable sites with more than one alternate allele and loci with minor allele frequencies less than 5%. Finally, we randomly selected a single SNP per every 1000 bp to increase the independence of loci used in subsequent analyses. Additional parameter settings for these analyses are available at Dryad [55].

### Spatial genetic structure

Analyses were performed on the full set of all eleven populations as well as a subset of six populations of *O. p. princeps* sampled along semi-continuous talus habitat across the Ruby–East Humboldt mountain range in eastern Nevada. To infer individual ancestry proportions (*q*) and the number of ancestral populations represented (*k*) in the dataset, we used a hierarchical Bayesian model (entropy v1.2) [60] that is based upon the commonly implemented model of structure [61, 62]. Entropy simultaneously estimates genotype probabilities and individual ancestry coefficients while incorporating genotype uncertainty inherent to low-to-medium coverage depth sequencing designs, as well as error associated with sequencing or subsequent mapping [60, 63, 64]. We ran five replicate MCMC chains for models ranging from *k* = 2 to *k* = 11 to determine the most probable *k* for the entire set of individuals, and for *k* = 2 to *k* = 8 for the Ruby-East Humboldt dataset. To speed the stabilization of MCMC chains, we initialized ancestry coefficients using cluster assignment probabilities generated from *k*-means clustering and linear discriminant analysis of principal component scores using the *mass* package in R v3.6.3 [65, 66]. All MCMC chains were run for 40,000 steps following 10,000-step burn-ins and thinning for one out of ten steps. Deviance Information Criterion (DIC) was used for model comparison. We further summarized patterns and levels of population genetic variation across all samples using the estimated genotype probabilities produced by entropy as the input variables for principal component analysis (PCA) performed using the *prcomp* function in R. The matrix of genotype probabilities is available at Dryad [55].

To evaluate the potential for population genetic structure across a range of geographic scales, we conducted separate PCA analyses on: (1) the full set of 171 individuals; (2) the subset of individuals sampled from the Ruby-East Humboldt Mountains in Nevada; (3) the subset of individuals from California (Bodie and Pipet Tarn); and (4) the subset of individuals sampled from Montana (Emerald Lake and Swan Creek). PCA is an effective model-free tool for illustrating spatial genetic structure across a continuum of differentiation, including among weakly differentiated populations [67–70], where even axes explaining lower levels of variance can reveal spatial genetic structure and population identifiability [71]. We further summarized patterns of genetic differentiation based on allele frequency variation by calculating Nei’s genetic distance (*D*) [72] for each pairwise comparison among populations.

### Geographic and environmental predictors of spatial genetic structure

To quantify the relative importance of climatic and geographic factors as predictors of spatial genetic structure, we implemented a series of multiple regressions on distance matrices (MRMs) within a model comparison framework. Separate analyses were conducted for both the entire eleven population dataset and the subset of six populations from the Ruby Mountains and East Humboldt Range. Haversine geographic distances between sampling locations were calculated based on the midpoints of latitude and longitude for each sampling region using the *fossil* package in R [73], whereas elevational distance was defined as the absolute difference in elevation between sites. Climatic variation was characterized for sampling localities using PCA based on 19 climatic variables downloaded for each site (1 km^2^ scale; https://www.worldclim.org) [74]. For each of the first two principal components (PCs) from the climate PCA (proportion of variance explained: full model = 46.9% and 33.8%; Ruby-East Humboldt subset = 60.0% and 31.0%) climatic distance among sites was calculated using the *dist* function in R (hereafter referred to as the Climate 1 and Climate 2 predictor variables). MRMs were performed using the *ecodist* package in R [75, 76], with Nei’s *D* as the response variable. All possible combinations of MRMs including 1, 2, 3, and 4 predictor variables (geographic distance, elevational distance, Climate 1 distance, Climate 2 distance) were analyzed.

### Quantifying levels of genetic diversity

We estimated nucleotide diversity (*π*) [77] and Watterson’s theta (θ_W_) [78] with ANGSD v0.921 [79] following the empirical Bayes method described by Korneliussen et al. [80]. Diversity metrics were calculated for each population independently using individual.bam files. First, site allele frequency likelihoods were estimated using the "-doSAF 1" command and specifying the samtools genotype likelihood model. Next, the folded site frequency spectrum (SFS) was estimated based on allele frequencies using the "realSFS" function. *π* and *θ*_W_ were calculated with the "doThetas 1" command using the SFS likelihoods as priors for each site. The "thetaStat" command was used to summarize values for each scaffold, and mean *π* and *θ*_W_ across scaffolds were calculated in R.

We also estimated Tajima’s *D* [81] using ANGSD. Tajima’s *D* is based on the difference between *π* and *θ*_W_, which are expected to be equivalent for populations at mutation-drift equilibrium [82]. However, these estimates of *θ* inherently differ in their response to selection or demographic events: *π* > *θ*_W_ when the SFS is skewed towards common variants (positive Tajima’s *D*), whereas *π* < *θ*_W_ when the SFS is skewed towards rare variants (negative Tajima’s *D*) [81]. During a bottleneck, low-frequency variants are disproportionately lost from a population [83], resulting in a SFS with more common variants than expected (positive Tajima’s *D*). Following a bottleneck, a population can rapidly expand, resulting in an increased number of mutations per generation and thus a SFS containing more rare variants than expected (negative Tajima’s *D*). It is worth noting that this is a broad oversimplification of the effects of a bottleneck on Tajima’s *D*, and that the resulting estimates can be strongly influenced by population size, the strength of the bottleneck, the time since the bottleneck, and the rate of subsequent population growth [82, 84–86]. For each population, Tajima’s *D* was calculated for 50 kbp windows across the genome (50 kbp step size) using the estimates of *π* and *θ*_W_ generated above. The mean value of Tajima’s *D* across all windows was calculated in R.

## Supplementary Information

**Additional file 1: Table S1.** Five replicate entropy (Gompert et al. 2014) chains were run for *k* = 2–11 for the full dataset of all pika populations. **Table S2.** Five replicate entropy (Gompert et al. 2014) chains were run for *k* = 2–8 for the Nevada subset of pika populations. **Table S3.** The loadings of bioclimatic variables onto the first two PCs for all 11 populations. **Table S4.** The loadings of bioclimatic variables onto the first two PCs for the six Nevadan populations. **Table S5.** Sample sizes (*N*) and estimates of genetic diversity for each population.

**Additional file 2: Fig. S1.** Pairwise comparisons of Nei’s *D* (Nei 1972) based on allele frequencies for each sampled pika population. The distribution of Nei’s *D* for all pairwise comparisons is represented by a heat map with warmer colors indicating greater genome-wide genetic differentiation. Figure created by authors KBK and JPJ.

**Additional file 3: Fig. S2.** The relationships among the four variables (elevational distance, geographic distance, (Climate 1 Distance, Climate 2 distance) used to predict genetic distance (see Fig. 6; Tables 2 and 3 in the main text) are depicted for all populations (All; upper triangle) and the subset of Nevadan populations (NV; lower triangle). Figure created by author JPJ.

## Data Availability

A directory containing the compressed individual.fastq files and a matrix of genotype probabilities used for downstream analyses can be found at Dryad Digital Repository 10.5061/dryad.mcvdncjww.

## Abbreviations

ddRADseq: Double digest restriction-site associated DNA sequencing
DIC: Deviance information criterion
GBS: Genotyping-by-sequencing
MRM: Multiple regression on distance matrices
PC: Principal component
PCA: Principal component analysis
SNP: Single nucleotide polymorphism
