# Supplementary material for: Genomic variation in the American pika: signatures of geographic isolation and implications for conservation
Source: BMC Ecol Evol. 2021 Jan 21;21:2. doi: 10.1186/s12862-020-01739-9 (PMC7853312; doi:10.1186/s12862-020-01739-9)
Supplement: Supplementary file 1 — Additional file 1: Table S1. Five replicate entropy (Gompert et al. 2014) chains were run for k = 2–11 for the full dataset of all pika populations. Table S2. Five replicate entropy (Gompert et al. 2014) chains were run for k = 2–8 for the Nevada subset of pika populations. Table S3. The loadings of bioclimatic variables onto the first two PCs for all 11 populations. Table S4. The loadings of bioclimatic variables onto the first two PCs for the six Nevadan populations. Table S5. Sample sizes (N) and estimates of genetic diversity for each population. [file 12862_2020_1739_MOESM1_ESM.docx]

Supplementary information

Genomic variation in the American pika: signatures of geographic isolation and implications for conservation

Kelly B. Klingler, Joshua P. Jahner, Thomas L. Parchman, Chris Ray, and Mary M. Peacock

| **Table S1.** Five replicate entropy (Gompert et al. 2014) chains were run for *k*=2-11 for the full dataset of all pika populations. Lower deviance information criterion (DIC) values are consistent with better model fit. | | |
| --- | --- | --- |
| *k* | Mean DIC | S.D. DIC |
| 2 | 10,809,248 | 277,181 |
| 3 | 5,362,799 | 72,362 |
| 4 | 4,911,094 | 47,217 |
| 5 | 72,468,249 | 151,098,403 |
| 6 | 193,643,277 | 180,334,477 |
| 7 | 210,382,149 | 95,748,238 |
| 8 | 304,346,501 | 70,951,197 |
| 9 | 217,698,526 | 108,368,336 |
| 10 | 215,534,591 | 255,909,699 |
| 11 | 219,086,067 | 156,532,153 |

| **Table S2.** Five replicate entropy (Gompert et al. 2014) chains were run for *k*=2-8 for the Nevada subset of pika populations. Lower deviance information criterion (DIC) values are consistent with better model fit. | | |
| --- | --- | --- |
| *k* | Mean DIC | S.D. DIC |
| 2 | 3,068,298 | 109,280 |
| 3 | 120,255,738 | 34,988,199 |
| 4 | 140,093,137 | 61,393,237 |
| 5 | 20,500,554 | 7,535,693 |
| 6 | 16,726,008 | 17,317,034 |
| 7 | 164,093,762 | 172,665,891 |
| 8 | 200,835,936 | 126,441,297 |

| **Table S3.** The loadings of bioclimatic variables onto the first two PCs for all 11 populations. PCs 1 and 2 accounted for 46.9% and 33.8% of the variation in the climate data, respectively, and correspond to the Climate 1 and Climate 2 variables used in MRM analyses. Loadings > 0.30 or < -0.30 are bolded. | | |
| --- | --- | --- |
| Bioclimatic variable | PC 1 | PC 2 |
| Annual mean temperature (1) | 0.2843 | 0.1869 |
| Mean diurnal range (2) | 0.2678 | -0.1782 |
| Isothermality (3) | 0.2458 | -0.2268 |
| Temperature seasonality (4) | -0.0128 | **0.3518** |
| Max temperature of the warmest month (5) | **0.3256** | 0.0261 |
| Min temperature of the coldest month (6) | 0.0963 | 0.2535 |
| Temperature annual range (7) | 0.2948 | -0.0894 |
| Mean temperature of the wettest quarter (8) | -0.0648 | 0.2050 |
| Mean temperature of the driest quarter (9) | 0.2264 | 0.0167 |
| Mean temperature of the warmest quarter (10) | 0.2510 | 0.2302 |
| Mean temperature of the coldest quarter (11) | **0.3150** | 0.1036 |
| Annual precipitation (12) | -0.2685 | -0.1726 |
| Precipitation of the wettest month (13) | -0.1408 | **-0.3168** |
| Precipitation of the driest month (14) | -0.2838 | 0.2015 |
| Precipitation seasonality (15) | 0.0967 | **-0.3529** |
| Precipitation of the wettest quarter (16) | -0.1366 | **-0.3269** |
| Precipitation of the driest quarter (17) | -0.2972 | 0.1697 |
| Precipitation of the warmest quarter (18) | -0.2788 | 0.1213 |
| Precipitation of the coldest quarter (19) | -0.0201 | **-0.3654** |

| **Table S4.** The loadings of bioclimatic variables onto the first two PCs for the six Nevadan populations. PCs 1 and 2 accounted for 60.0% and 31.0% of the variation in the climate data, respectively, and correspond to the Climate 1 and Climate 2 variables used in MRM analyses. Loadings > 0.30 or < -0.30 are bolded. | | |
| --- | --- | --- |
| Bioclimatic variable | PC 1 | PC 2 |
| Annual mean temperature (1) | 0.2716 | 0.1613 |
| Mean diurnal range (2) | 0.1837 | -0.2360 |
| Isothermality (3) | 0.0668 | -0.2768 |
| Temperature seasonality (4) | 0.2896 | 0.0651 |
| Max temperature of the warmest month (5) | 0.2751 | 0.1479 |
| Min temperature of the coldest month (6) | 0.1612 | **0.3402** |
| Temperature annual range (7) | 0.2631 | -0.1545 |
| Mean temperature of the wettest quarter (8) | 0.2350 | 0.1473 |
| Mean temperature of the driest quarter (9) | 0.2704 | 0.1674 |
| Mean temperature of the warmest quarter (10) | 0.2704 | 0.1674 |
| Mean temperature of the coldest quarter (11) | 0.2394 | 0.2362 |
| Annual precipitation (12) | -0.1856 | 0.2908 |
| Precipitation of the wettest month (13) | -0.0751 | **0.3827** |
| Precipitation of the driest month (14) | -0.2818 | 0.1142 |
| Precipitation seasonality (15) | 0.2749 | 0.1263 |
| Precipitation of the wettest quarter (16) | -0.0174 | **0.3827** |
| Precipitation of the driest quarter (17) | -0.2298 | 0.2374 |
| Precipitation of the warmest quarter (18) | -0.2298 | 0.2374 |
| Precipitation of the coldest quarter (19) | -0.2711 | 0.1527 |

| **Table S5.** Sample sizes (*N*) and estimates of genetic diversity for each population. Nucleotide diversity (𝜋, Tajima 1983), Watterson’s theta (𝜃_W_, Watterson 1975), and Tajima’s *D* (Tajima 1989) were calculated in ANGSD (Korneliussen et al. 2013, 2014). Means and 95% confidence intervals are presented for each estimate. *O. p. schisticeps*: California (CA) populations; *O. p. saxatilis*: Colorado (CO) population; *O. p. princeps*: Montana (MT) and Nevada (NV) populations. | | | | | | | | | | |
| --- | --- | --- | --- | --- | --- | --- | --- | --- | --- | --- |
| Pop | *N* | 𝜋 | 𝜋 lower | 𝜋 upper | 𝜃_W_ | 𝜃_W_ lower | 𝜃_W_ upper | *D* | *D* lower | *D* upper |
| Bodie, CA | 15 | 0.00147 | 0.00146 | 0.00148 | 0.00110 | 0.00109 | 0.00110 | 0.811 | 0.805 | 0.817 |
| Pipet Tarn, CA | 20 | 0.00193 | 0.00192 | 0.00194 | 0.00168 | 0.00168 | 0.00169 | 0.349 | 0.343 | 0.355 |
| West Knoll, CO | 21 | 0.00253 | 0.00252 | 0.00254 | 0.00214 | 0.00213 | 0.00214 | 0.478 | 0.472 | 0.484 |
| Emerald Lake, MT | 21 | 0.00273 | 0.00272 | 0.00274 | 0.00216 | 0.00215 | 0.00216 | 0.712 | 0.706 | 0.718 |
| Swan Creek, MT | 7 | 0.00276 | 0.00275 | 0.00277 | 0.00239 | 0.00238 | 0.00240 | 0.475 | 0.470 | 0.480 |
| Overland Lake, NV (RM) | 17 | 0.00091 | 0.00091 | 0.00091 | 0.00066 | 0.00066 | 0.00067 | 0.767 | 0.760 | 0.774 |
| Island Lake, NV (RM) | 16 | 0.00087 | 0.00086 | 0.00087 | 0.00066 | 0.00066 | 0.00066 | 0.585 | 0.578 | 0.592 |
| Hidden Lake, NV (RM) | 15 | 0.00082 | 0.00082 | 0.00083 | 0.00062 | 0.00062 | 0.00063 | 0.495 | 0.487 | 0.503 |
| Week’s Creek, NV (EH) | 10 | 0.00066 | 0.00066 | 0.00067 | 0.00053 | 0.00053 | 0.00054 | 0.326 | 0.319 | 0.333 |
| Lizzie’s Basin, NV (EH) | 10 | 0.00066 | 0.00065 | 0.00066 | 0.00057 | 0.00057 | 0.00057 | 0.240 | 0.233 | 0.246 |
| Smith Lake, NV (EH) | 19 | 0.00077 | 0.00077 | 0.00077 | 0.00053 | 0.00053 | 0.00054 | 0.799 | 0.792 | 0.807 |
