# Supplementary figures and images for: Genomic variation in the American pika: signatures of geographic isolation and implications for conservation
Source: BMC Ecol Evol. 2021 Jan 21;21:2. doi: 10.1186/s12862-020-01739-9 (PMC7853312; doi:10.1186/s12862-020-01739-9)

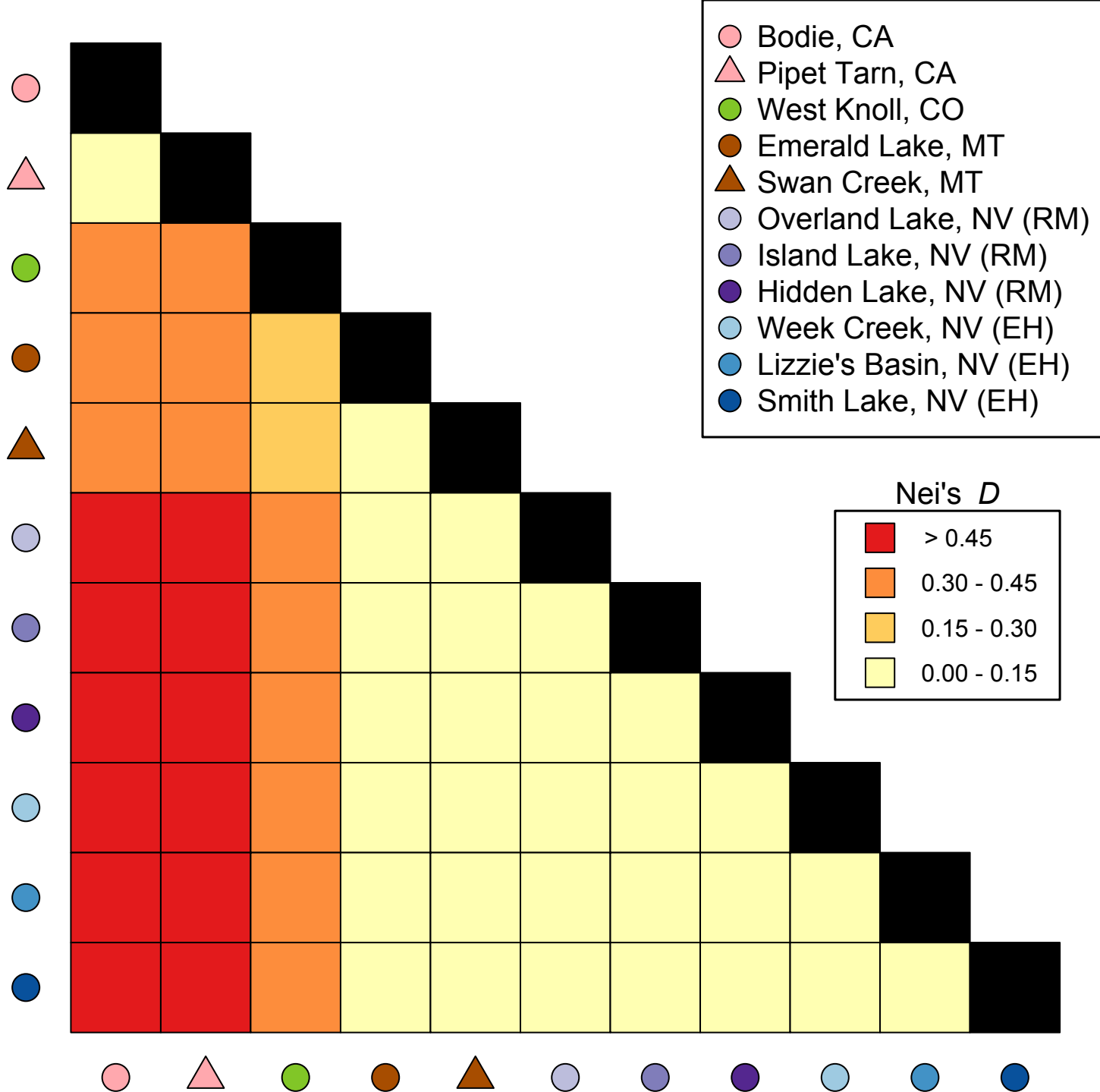

Supplement: Supplementary file 2 — Additional file 2: Fig. S1. Pairwise comparisons of Nei’s D (Nei 1972) based on allele frequencies for each sampled pika population. The distribution of Nei’s D for all pairwise comparisons is represented by a heat map with warmer colors indicating greater genome-wide genetic differentiation. Figure created by authors KBK and JPJ. [file 12862_2020_1739_MOESM2_ESM.pdf]

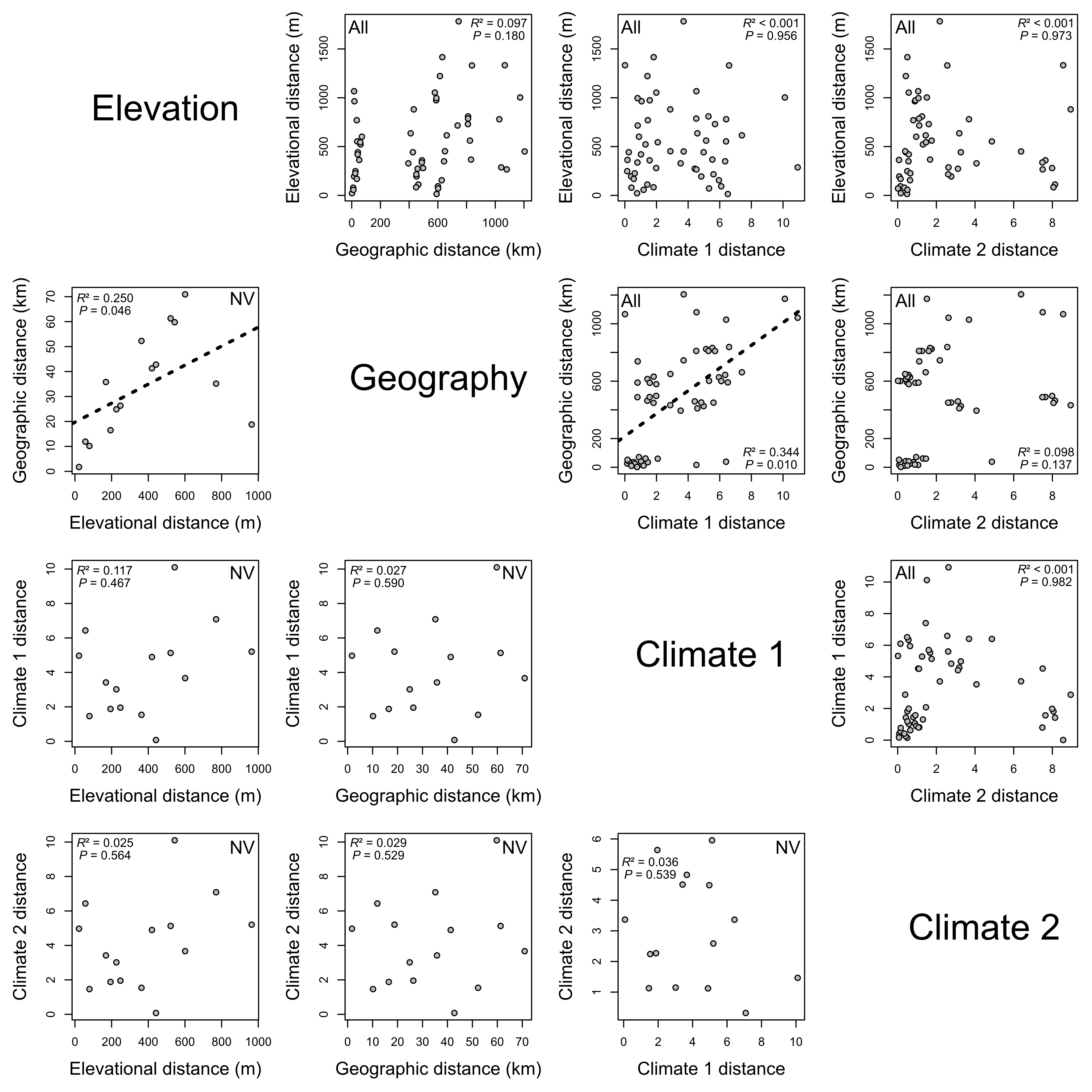

Supplement: Supplementary file 3 — Additional file 3: Fig. S2. The relationships among the four variables (elevational distance, geographic distance, (Climate 1 Distance, Climate 2 distance) used to predict genetic distance (see Fig. 6; Tables 2 and 3 in the main text) are depicted for all populations (All; upper triangle) and the subset of Nevadan populations (NV; lower triangle). Figure created by author JPJ. [file 12862_2020_1739_MOESM3_ESM.tiff]
